# Comparative study of the therapeutic effects of different medications on DM-associated dry eye syndrome after vitrectomy

**DOI:** 10.3389/fmed.2026.1795145

**Published:** 2026-05-12

**Authors:** Fang Ge, Min Du, Yuan Tao, Yanjuan Sheng, Xu Wang, Xing Du

**Affiliations:** 1 Department of Ophthalmology, Jinan Second People’s Hospital, Jinan, Shandong, China; 2 Department of Hospital Infection Management, Jinan Second People’s Hospital, Jinan, Shandong, China

**Keywords:** ciclosporin, diabetes mellitus, dry eye disease, sodium hyaluronate, vitrectomy

## Abstract

No prior studies have compared ciclosporin (CsA) and sodium hyaluronate (SH) eye drops for treating diabetes mellitus-associated dry eye syndrome (DMDES) after vitrectomy. To evaluate their relative efficacy and inform postoperative management, we conducted a retrospective comparative cohort study of 204 patients with DMDES who underwent vitrectomy for diabetic vitreous hemorrhage. Postoperatively, 104 patients received 0.05% CsA eye drops and 100 cases received SH eye drops. Noninvasive breakup time (NIBUT), tear meniscus height (TMH), Schirmer *I* test (SIT), and corneal fluorescein staining score (CFSS) were assessed preoperatively and at 1 and 3 months postoperatively. Longitudinal analysis of clinical outcomes using Linear Mixed-Effects Model (LMM) (for NIBUT, TMH, and SIT) and Generalized Estimating Equations (GEE) model (for CFSS). The LMM revealed a significant group × time interaction in favor of the CsA group for NIBUT, TMH, SIT. Specifically, compared to the SH group, the CsA group was associated with greater relative improvements at 1 month for NIBUT, TMH, and SIT. Covariates including age and gender did not show significant main effects on these outcomes (all *p* > 0.05). The GEE model revealed significant, negative interaction terms for the CsA group at 1 month and 3 months postoperatively. A lower CFSS indicates less corneal epithelial damage, these negative coefficients suggest that the CsA group showed a decrease in corneal staining scores compared to the SH group over the follow-up period. Age and gender were not significantly associated with CFSS changes (all *p* > 0.05). Overall, 0.05% CsA eye drops were associated with improved tear film quality and stability after vitrectomy for DMDES, showed a trend of better relative improvement compared to SH eye drops.

## 1 Introduction

The global population of patients with diabetes mellitus (DM) is projected to reach 643 million by 2030 (1). With the continuous increase in the number of patients with diabetes, the prevalence of diabetes-related chronic ocular diseases has also risen, including diabetic retinopathy (DR) and dry eye disease (DED). DED is an ocular surface disorder characterized by tear film instability and hyperosmolarity, leading to inflammation and damage of the ocular surface (2). Its pathogenesis involves multiple factors, such as aging, autoimmune conditions, dysbiosis, environmental influences, use of visual display terminals, and surgical interventions (3, 4). The global prevalence of DED ranges from 5 to 34% (5–7), and the disease often results in ocular discomfort and visual impairment (3). According to the latest report of the Dry Eye Workshop (DEWS II), DM is a recognized risk factor for DED, and symptoms are more severe in patients with diabetes than in non-diabetic individuals (3). A 10-year longitudinal study demonstrated that approximately half of patients with diabetes also suffer from DED (8). Therefore, strengthening research on DM-associated dry eye syndrome (DMDES) is of substantial clinical importance. Currently, commonly used pharmacological treatments for DED include ciclosporin (CsA) eye drops and sodium hyaluronate (SH) eye drops (2). As an artificial tear substitute, SH eye drops are effective in alleviating the symptoms of dry eye (9). CsA eye drops can promote the secretion of tears and mucins, thereby improving tear film stability (10). Previous studies have compared the efficacy differences between diquafosol sodium eye drops and SH eye drops in treating patients with DMDES (11, 12). Currently, there is no comparative study on the efficacy of CsA eye drops and SH eye drops in treating DMDES after vitrectomy. To clarify the effects and explore more effective treatment methods, we have conducted research on this issue.

## 2 Materials and methods

### 2.1 General information

This retrospective comparative cohort study included 204 patients (204 eyes) who underwent vitrectomy for diabetic vitreous hemorrhage in the Department of Ophthalmology at Jinan Second People’s Hospital between January 2023 and September 2025. The patients ranged in age from 37 to 80 years, with a mean age of 57.69 ± 8.48 years. There were 112 male patients (112 eyes) and 92 female patients (92 eyes). Inclusion criteria were as follows: (1) a preoperative diagnosis of type 2 DM complicated by DR; (2) vitrectomy performed for vitreous hemorrhage; (3) a preoperative diagnosis of DED; and (4) complete preoperative and postoperative clinical data. Exclusion criteria included: (1) band-like echoes connected to the ocular wall within the vitreous cavity on B-scan ultrasonography; (2) lens opacity requiring cataract surgery; (3) intraoperative corneal epithelial injury; (4) lacrimal drainage system diseases; (5) entropion, trichiasis, eyelid skin laxity, or conjunctivochalasis; and (6) connective tissue diseases. During this period, a total of 614 vitrectomy surgeries were performed due to vitreous hemorrhage caused by diabetic retinopathy. Among them, there were 13 patients with type 1 diabetes, 101 patients without diagnosed DED, and 74 patients with incomplete data. Therefore, 426 patients met the inclusion criteria. Sixty-five patients met exclusion criterion 1, 105 patients met exclusion criterion 2, 14 patients met exclusion criterion 3, 11 patients met exclusion criterion 4, 25 patients met exclusion criterion 5, and 2 patients met exclusion criterion 6. Ultimately, 204 patients were included in the study. For the missing data in the study, this research adopted complete case analysis for processing, without data imputation. Only the cases with complete data were subjected to statistical analysis. Baseline demographic and clinical characteristics are summarized in Table 1. This study was conducted in accordance with the principles of the Declaration of Helsinki and its subsequent amendments. The study was approved by the Ethics Committee of Jinan Second People’s Hospital (No. JNEYE20230101). Ethics Committee of Jinan Second People’s Hospital waived the requirement for written informed consent.

**Table 1:** Comparison of baseline characteristics between CsA group and SH group.

Parameter	CsA group (104 eyes)	SH group (100 eyes)	*P*	SMD
Age (years)	56.76 ± 8.33	58.65 ± 8.57	0.112^a^	0.224
Gender (Male: Female)	58:46	54:46	0.800^b^	0.036
Eye (Left: Right)	56:48	53:47	0.900^b^	0.054
NIBUT (s)	6.51 ± 1.55	6.86 ± 1.62	0.117^a^	0.220
TMH (um)	155.95 ± 32.57	160.57 ± 43.11	0.389^a^	0.121
SIT (mm/5 min)	5.50 ± 1.50	5.60 ± 2.24	0.712^a^	0.052
CFSS	1.0(0.0, 1.0)	1.0(0.0, 2.0)	0.603^c^	0.134

^a^Independent samples *t*-test, ^b^*χ*^2^ test, ^c^Mann–Whitney *U* test. NIBUT, non-invasive tear film breakup time, unit: second; TMH, tear meniscus height, unit, um; SIT, Schirmer *I* test, unit: mm/5 min; CFSS, corneal fluorescein staining score. SMD, Standardized Mean Difference.

### 2.2 Surgical procedure

All surgeries were performed by the same surgeon. Retrobulbar anesthesia was administered using 2% lidocaine injection. The conjunctival sac was irrigated with povidone–iodine solution, and balanced salt solution (BSS) was used intraoperatively to maintain corneal hydration. All procedures were performed using a Constellation vitrectomy system (Constellation Vitrectomy, Alcon, United States). Three 25-gauge transscleral ports were created, and the vitreous body in both the anterior and posterior segments was completely removed. Retinal laser photocoagulation was performed as indicated. At the end of surgery, scleral incisionsites were examined for leakage, and conjunctival electrocautery was applied when necessary. Postoperatively, all patients received moxifloxacin eye drops (Vigamox, Alcon, United States) four times daily for 3 weeks and 1% prednisolone acetate eye drops (Pred Forte, Allergan, United States) four times daily for 3 weeks. Compound tropicamide eye drops (Mydrin-P, Santen, Japan) were administered once nightly for 1 week. Patients in the CsA group received 0.05% CsA eye drops (Shenyang Sinqi Pharmaceutical Co., Ltd.) twice daily for 3 months, whereas patients in the SH group received 0.3% SH eye drops (Santen Pharmaceutical Co., Ltd.) four times daily for 3 months.

### 2.3 Examination methods

Patients underwent examinations 1 day preoperatively and at 1 and 3 months postoperatively, including noninvasive breakup time (NIBUT), tear meniscus height (TMH), Schirmer *I* test (SIT), and corneal fluorescein staining score (CFSS). All examinations were performed by the same physician under identical environmental conditions and were conducted 2 h after instillation of eye drops.

#### 2.3.1 Noninvasive breakup time

NIBUT was measured using a Keratograph 5 M (OCULUS, Germany) ocular surface analyzer. Each measurement was performed three times, and the mean value was recorded as the average tear film breakup time (13).

#### 2.3.2 Central tear meniscus height

Central TMH was measured using the Keratograph 5 M ocular surface analyzer. Images of the tear meniscus were captured, and the built-in measurement tool was used to measure the central TMH directly below the pupil. Measurements were expressed in micrometers (um) (13, 14).

#### 2.3.3 Schirmer *I* test

The Schirmer *I* test (SIT) was performed without topical anesthesia. After folding the Schirmer test strip 5 mm from the tip, it was placed in the conjunctival sac at the junction of the middle and lateral one-third of the lower eyelid, with the outer end hanging freely. After the patient closed the eyes for 5 min, the strip was removed intact. The length of the moistened area on the strip, measured in millimeters (mm), represented tear secretion volume (13).

#### 2.3.4 Corneal fluorescein staining score

CFSS assessment was initiated after completion of the SIT. Patients were instructed to blink 3–4 times to allow uniform distribution of fluorescein over the ocular surface. Corneal epithelial staining was then examined under cobalt blue light. Scoring was performed according to published criteria (15). The cornea was divided into five regions: temporal, superior, nasal, inferior, and central. Each region was scored based on the extent of fluorescein staining: 0 points for no staining, 1 point for punctate staining, 2 points for patchy staining, and 3 points for confluent staining. The total score was calculated as the sum of all five regions, with a maximum score of 15.

### 2.4 Diagnostic criteria

According to the 2020 Chinese Expert Consensus on the Diagnosis and Examination of Dry Eye Disease (16), DED was diagnosed based on the following criteria: (1) the presence of at least one subjective symptom, such as ocular dryness, foreign body sensation, burning sensation, fatigue, ocular redness, or visual fluctuation, accompanied by NIBUT ≤ 10 s or SIT ≤ 5 mm/5 min; or (2) the presence of dry eye–related symptoms with 10 s < NIBUT < 12 s or 5 mm/5 min < SIT ≤ 10 mm/5 min, along with positive fluorescein staining. Patients meeting either of the above criteria were diagnosed with DED.

### 2.5 Statistical analysis

Statistical analysis was performed using SPSS version 23.0, with *p* < 0.05 considered statistically significant. Data normality was assessed using the Shapiro–Wilk test. Normally distributed continuous variables were expressed as mean ± standard deviation (Mean ± SD). Age data in both groups followed a normal distribution and were compared using independent-samples t-tests. Gender and the tested eye (left/right) were treated as categorical variables, expressed as frequencies, and compared using the chi-square test (*χ*^2^ test). The preoperative and postoperative NIBUT, TMH, and SIT values followed normal distributions. We employed a Linear Mixed-Effects Model (LMM) for the longitudinal analysis of these repeated continuous measures. Residual diagnostics (Q–Q plots and scatter plots of residuals versus fitted values) were visually inspected to verify the assumptions of normality and homoscedasticity, which were deemed acceptable. For this LMM structure, ‘Treatment Group’, ‘Time Point’ (including baseline), and the ‘Group × Time’ interaction were included as fixed effects. ‘Patient ID’ was incorporated as a random intercept to account for within-subject correlation. To address potential case-mix differences, we adjusted for ‘Age’ and ‘Gender’ as baseline covariates. The CFSS data were not normally distributed and were expressed as median (P25, P75). Given the ordinal and bounded nature of the CFSS clinical grading, a Generalized Estimating Equations (GEE) model was utilized as a reasonable pragmatic approximation to handle the longitudinal within-subject correlation. The CFSS data were modeled using a Poisson distributional family with a log link function, and an exchangeable working correlation structure was specified to appropriately manage within-subject dependencies over time. Similar to the LMM, ‘Treatment Group’, ‘Time Point’, and the ‘Group × Time’ interaction were included in the GEE model, with ‘Age’ and ‘Gender’ adjusted as covariates. We designated NIBUT at 3 months postoperatively as the primary endpoint, given its robust ability to evaluate tear film stability. TMH, SIT, and CFSS were classified as secondary endpoints. To robustly address the multiplicity of evaluating four distinct clinical outcomes, a strict Bonferroni correction was applied. Consequently, while nominal statistical significance was initially evaluated at a two-sided *p* < 0.05, the stringent adjusted threshold for definitive statistical significance in the longitudinal models was set at *p* < 0.0125 (calculated as 0.05/4).

## 3 Results

### 3.1 Baseline characteristics

The baseline characteristics of patients in the CsA and SH groups, including age, gender, left/right eye, and preoperative values of NIBUT, TMH, SIT, and CFSS, are summarized in Table 1. There were no statistically significant differences between the two groups for any of these variables (*p* > 0.05). To formally evaluate the balance of baseline covariates between the two treatment groups without the sample-size dependency of traditional hypothesis testing, we calculated the Standardized Mean Difference (SMD). The SMDs for gender (0.036), TMH (0.121), SIT (0.052), and CFSS (0.134) were all well below 0.20, indicating excellent comparability. However, minor imbalances were detected for age (SMD = 0.224) and baseline NIBUT (SMD = 0.220). To rigorously prevent these slight imbalances from confounding the comparative efficacy analysis, age and gender were explicitly incorporated as covariates for adjustment in all subsequent longitudinal models (LMM and GEE).

### 3.2 Changes in NIBUT, TMH, SIT, and CFSS before and after operation

**Figure 1:**
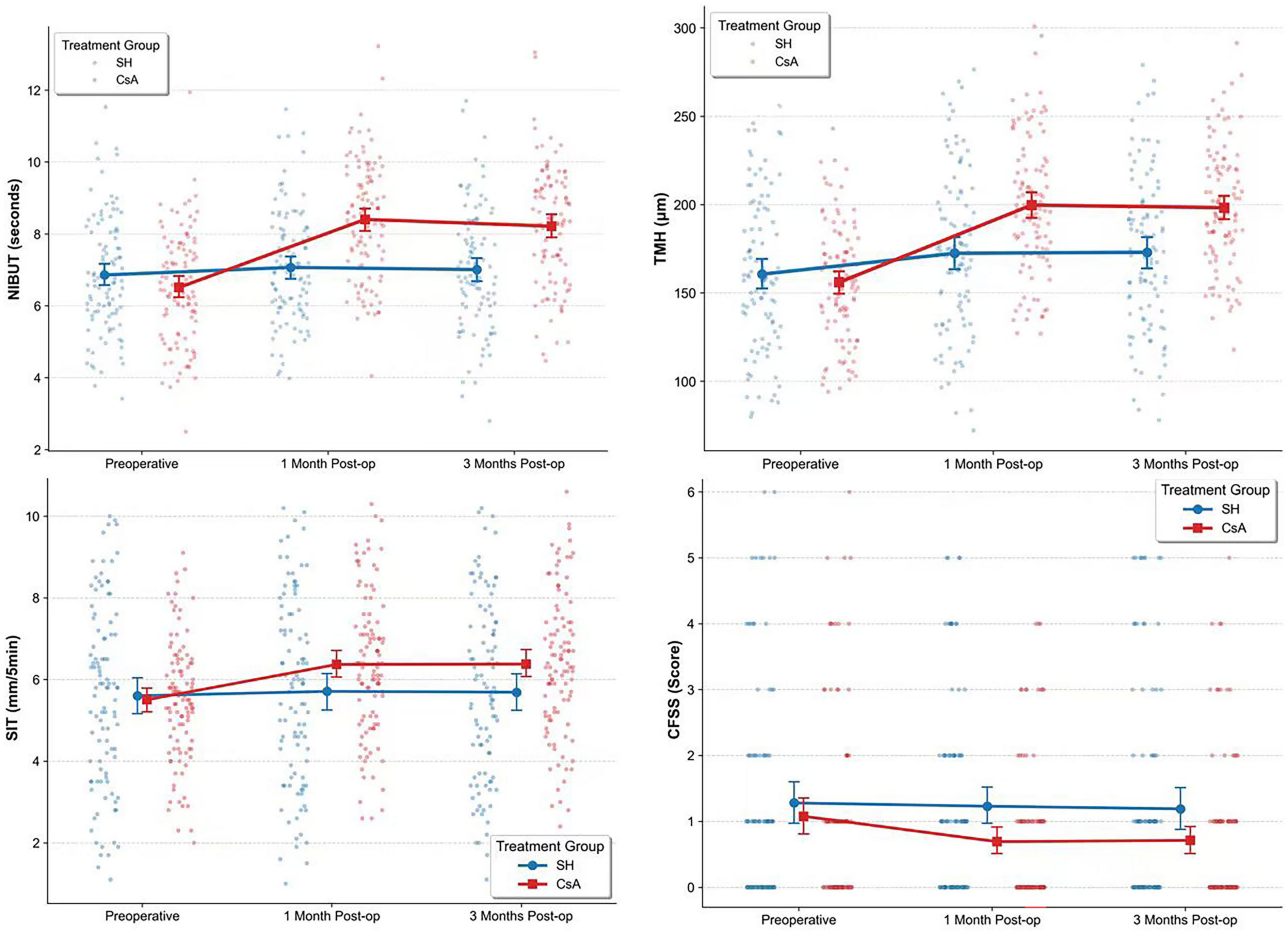
The NIBUT, TMH, SIT and CFSS changes of each time point. NIBUT: noninvasive breakup time, unit: second. TMH: tear meniscus height, unit: um. SIT, Schirmer *I* test, unit: mm/5 min. CFSS, corneal fluorescein staining score. Red point: data of the CsA group. Blue point: data of the SH group.

The preoperative and postoperative (1 and 3 months) values of NIBUT, TMH, SIT, and CFSS in the CsA and SH groups are presented in Table 2 and Figure 1. At preoperative baseline, no significant differences were observed in NIBUT, TMH, and SIT between the CsA group and SH group (all *p* > 0.05). However, as shown in Table 3 and Figure 1, the LMM revealed a significant group × time interaction in favor of the CsA group for all three metrics. Specifically, compared to the SH group, the CsA group suggested a relatively good improvements at 1 month for NIBUT (Estimate = 1.683, 95% CI: 1.529 to 1.837, *p* < 0.001), TMH (Estimate = 31.918, 95% CI: 27.905 to 35.931, *p* < 0.001), and SIT (Estimate = 0.696, 95% CI: 0.454 to 0.938, *p* < 0.001). This relatively steep trajectory of recovery in the CsA group was sustained at 3 months postoperatively (all interaction *p* < 0.001). Covariates including age and gender did not show significant main effects on these outcomes (all *p* > 0.05), indicating that relatively good recovery trajectories in the CsA group were independent of these demographic factors.

**Table 2:** The data of NIBUT, TMH, SIT and CFSS at each time point before and after operation.

Clinical outcome	Group	Pre-operation	1 month Post-op	3 months Post-op
NIBUT	CsA	6.51 ± 1.56	8.40 ± 1.63	8.21 ± 1.65
	SH	6.86 ± 1.62	7.07 ± 1.57	7.01 ± 1.70
TMH	CsA	155.96 ± 32.79	199.69 ± 38.73	198.19 ± 34.39
	SH	160.57 ± 43.11	172.39 ± 45.50	172.97 ± 44.65
SIT	CsA	5.50 ± 1.49	6.37 ± 1.75	6.38 ± 1.74
	SH	5.60 ± 2.24	5.71 ± 2.25	5.69 ± 2.26
CFSS	CsA	1.0(0.0, 1.0)	0.0(0.0, 1.0)	0.0(0.0, 1.0)
	SH	1.0(0.0, 2.0)	1.0(0.0, 2.0)	1.0(0.0, 1.75)

NIBUT, non-invasive breakup time, unit: second; TMH, tear meniscus height, unit: um; SIT, Schirmer *I* test, unit: mm/5 min; CFSS, corneal fluorescein staining score.

**Table 3:** LMM (for NIBUT, TMH, and SIT) and GEE (for CFSS) results for evaluating treatment and time effects.

Clinical outcome	Effect/variable	Estimate (Coef.)	SE	*Z*/*T*-value	95% CI	*p*-value
NIBUT (LMM)	Intercept	5.906	0.797	7.41	4.344 to 7.469	<0.001
	Group (CsA vs. SH)	−0.329	0.228	−1.442	−0.775 to 0.118	0.149
	Time (1 Month vs. Preop)	0.212	0.056	3.764	0.101 to 0.322	<0.001
	Time (3 Months vs. Preop)	0.149	0.056	2.65	0.039 to 0.259	0.008
	Group × Time (CsA × 1 Month)	1.683	0.079	21.375	1.529 to 1.837	<0.001
	Group × Time (CsA × 3 Months)	1.556	0.079	19.758	1.401 to 1.710	<0.001
	Age	0.014	0.013	1.023	−0.012 to 0.039	0.306
	Gender (Male vs. Female)	0.295	0.223	1.322	−0.142 to 0.732	0.186
TMH (LMM)	Intercept	131.529	19.538	6.732	93.235 to 169.822	<0.001
	Group (CsA vs. SH)	−3.376	5.6	−0.603	−14.352 to 7.599	0.547
	Time (1 Month vs. Preop)	11.82	1.462	8.086	8.955 to 14.686	<0.001
	Time (3 Months vs. Preop)	12.397	1.462	8.481	9.532 to 15.262	<0.001
	Group × Time (CsA × 1 Month)	31.918	2.047	15.59	27.905 to 35.931	<0.001
	Group × Time (CsA × 3 Months)	29.843	2.047	14.576	25.830 to 33.855	<0.001
	Age	0.575	0.323	1.779	−0.059 to 1.209	0.075
	Gender (Male vs. Female)	−8.71	5.466	−1.593	−19.423 to 2.004	0.111
SIT (LMM)	Intercept	4.775	0.983	4.859	2.849, 6.701	<0.001
	Group (CsA vs. SH)	−0.072	0.279	−0.259	−0.620, 0.475	0.796
	Time (1 Month vs. Preop)	0.108	0.058	1.866	−0.005, 0.221	0.062
	Time (3 Months vs. Preop)	0.087	0.058	1.503	−0.026, 0.200	0.133
	Group × Time (CsA × 1 Month)	0.757	0.081	9.343	0.598, 0.916	<0.001
	Group × Time (CsA × 3 Months)	0.787	0.081	9.709	0.628, 0.946	<0.001
	Age	0.014	0.016	0.853	−0.018, 0.046	0.394
	Gender (Male vs. Female)	0.023	0.275	0.085	−0.516, 0.562	0.933
CFSS (GEE)	Intercept	−0.2346	0.558	−0.421	−1.328, 0.859	0.674
	Group (CsA vs. SH)	−0.1659	0.178	−0.934	−0.514, 0.182	0.350
	Time (1 Month vs. Preop)	−0.0398	0.041	−0.963	−0.121, 0.041	0.336
	Time (3 Months vs. Preop)	−0.0729	0.031	−2.353	−0.134, −0.012	0.019
	Group × Time (CsA × 1 Month)	−0.4020	0.07	−5.726	−0.540, −0.264	<0.001
	Group × Time (CsA × 3 Months)	−0.3415	0.067	−5.126	−0.472, −0.211	<0.001
	Age	0.0060	0.009	0.657	−0.012, 0.024	0.511
	Gender (Male vs. Female)	0.2215	0.18	1.233	−0.131, 0.574	0.218

NIBUT, non-invasive breakup time, unit: second; TMH, tear meniscus height, unit: um; SIT, Schirmer I test, unit: mm/5 min; CFSS, corneal fluorescein staining score. SH group and Preoperative time point were used as reference categories. SE, Standard Error; CI, Confidence Interval. The interaction term (Group × Time) represents the differential treatment effect of CsA over SH across the specified time points. In the linear mixed-effects model, the SH group and the preoperative time point were used as reference categories. Therefore, the main effect of ‘Group (CsA vs. SH)’ evaluates the estimated difference between the two treatments at the preoperative baseline.

As shown in Table 3 and Figure 1, The GEE analysis suggested no significant difference in baseline CFSS between the two groups (*p* = 0.350). However, the model revealed significant, negative interaction terms for the CsA group at 1 month (Coefficient = −0.4020, 95% CI: −0.540 to −0.264, *p* < 0.001) and 3 months (Coefficient = −0.3415, 95% CI: −0.472 to −0.211, *p* < 0.001) postoperatively. Because lower CFSS indicates less corneal epithelial damage, these negative coefficients suggest that the CsA group experienced a relatively steep and more decline in corneal staining scores compared to the SH group over the follow-up period. Age and gender were not significantly associated with CFSS changes (all *p* > 0.05).

## 4 Discussion

Tear film quality is a critical determinant of postoperative ocular comfort. Previous studies have demonstrated that ocular discomfort after vitrectomy is closely associated with decreased tear film stability (17–19). This reduction in stability is related to multiple intraoperative factors, including prolonged ocular surface exposure, continuous irrigation with fluids, the use of povidone–iodine, and mechanical injury to the meibomian glands caused by eyelid speculums (20, 21). Chronic hyperglycemia, diabetic peripheral neuropathy, decreased insulin levels, microvascular lesions, and systemic hyperosmolar disturbances are recognized risk factors for DMDES (9). Many patients with DR require vitrectomy (22, 23). Consequently, patients with diabetes are more susceptible to developing dry eye after vitrectomy and may even experience complications such as delayed corneal epithelial healing and recurrent corneal epithelial erosion. As a result, increasing attention has been paid to tear film status in patients with DMDES following vitrectomy. In the present study, tear film changes before and after surgery were evaluated using NIBUT, TMH, SIT, and CFSS. The Keratograph 5 M ocular surface analyzer is an objective and comprehensive device for ocular surface assessment, with good repeatability and reliability (13, 24). It offers several advantages, including non-contact, non-invasive, and automated measurements, without the need for fluorescein staining. Because the mean breakup time provides a more comprehensive assessment of tear film stability than the first breakup time, the mean breakup time was selected as the NIBUT parameter in this study. In addition, the Keratograph 5 M system allows for accurate measurement of TMH (13).

We believe that corneal sensitivity and tear secretion are both reduced in patients with DM (25, 26). Decreased corneal sensitivity leads to diminished reflex tear secretion (27) and is also associated with a reduced blink rate, resulting in excessive tear evaporation and increased tear osmolarity (28). Elevated tear osmolarity activates stress signaling pathways in ocular surface immune cells, leading to the recruitment and activation of CD4^+^ T cells. These activated T cells proliferate and release proinflammatory cytokines (4, 29), which cause corneal epithelial damage and the loss of conjunctival goblet cells and mucins. The reduction in mucins further exacerbates tear film instability, thereby further increasing tear osmolarity, amplifying inflammation, and initiating a vicious cycle of ocular surface inflammation (4, 30, 31), ultimately triggering and aggravating the development of DMDES. In patients with DMDES, vitrectomy further exacerbates both the incidence and severity of dry eye. Therefore, early screening and management of DMDES are essential. Early control of ocular surface inflammation (3, 32) can interrupt this vicious cycle and alleviate discomfort in patients with DMDES.

CsA is a calcineurin inhibitor that enters cells and binds to cyclophilin, thereby inactivating calcineurin and inhibiting the dephosphorylation of nuclear factor of activated T cells (NFAT). This process downregulates the expression of inflammation-related genes, including *IL-2, IL-1β, IL-6, TNF-α, C1q,* and *IL-17A*, suppresses T-cell activation, and reduces the production and release of inflammatory mediators (2, 10, 33). Attenuation of ocular surface inflammation facilitates the restoration of conjunctival epithelial homeostasis, increases goblet cell density, and stimulates mucin secretion (34), thereby improving tear film stability (31, 34, 35). In addition, CsA alleviates eyelid margin inflammation, reduces meibomian gland obstruction, and increases meibomian gland secretion (36), leading to improvement in tear lipid abnormalities and reduced tear evaporation. Increased levels of mucins and lipids in the tear film improves tear film stability (3). Consequently, postoperative NIBUT in the CsA group was relatively increased than that in the SH group, consistent with the findings of Stonecipher et al. (37). By inhibiting T-cell activation and inflammatory mediator release (2, 10, 33), CsA reduces inflammation of the lacrimal and accessory lacrimal glands and restores the secretory function of lacrimal acinar cells (38), resulting in improved tear secretion. Moreover, CsA promotes tear secretion by modulating parasympathetic and trigeminal nerve pathways (39). Therefore, TMH and SIT values in the CsA group were relatively higher than those in the SH group, in agreement with the results reported by Chen et al. (40). Previous studies have also showed that CsA can restore cellular viability, promote wound healing, and mitigate hyperosmolarity-induced corneal damage (2). Accordingly, compared with the SH group, the CsA group had relatively less corneal staining and improved CFSS after surgery, which is consistent with the results of Xu et al.’s (36) study. In contrast, SH eye drops, as artificial tears, primarily alleviate dry eye–related discomfort (9). Studies have shown that SH eye drops can alleviate discomfort symptoms in patients with dry eye syndrome and are recommended as a choice for mild to moderate DED (41). However, as artificial tears, SH eye drops do not promote secretion or inhibit inflammation, so their effect may not be significant in patients with DMDES. Besides, given that all examinations in the present study were performed 2 h after eye drop instillation, the short-term lubricating effect of SH may have diminished, which likely explains the lack of significant improvement in NIBUT, TMH, SIT, and CFSS in the SH group.

In summary, our findings suggest that CsA eye drops may serve as a beneficial therapeutic option associated with improved tear film stability, exhibit a relatively good recovery trend, and can improve tear film stability in DMDES patients after vitrectomy, which is consistent with the findings reported by Messmer et al. (32). Although this study fills an important gap in the evidence regarding the use of CsA for the treatment of DMDES, several limitations should be acknowledged. Although we adjusted the major demographic factors (age, gender), we recognize that as a retrospective cohort study, we cannot capture and adjust every potential unmeasured confounding factor, such as the onset age of diabetes, disease duration, normal blood glucose fluctuation level and systemic medication history, which may subtly affect the tear film state. Furthermore, this study is a single-center study design, with a relatively small sample size and short follow-up duration. Therefore, larger-scale, multicenter, prospective randomized controlled trials are warranted to provide more robust and definitive evidence.

## Data Availability

The original contributions presented in the study are included in the article/supplementary material, further inquiries can be directed to the corresponding authors.
